# Traumatic Disruption of Profunda Femoris Artery Branch Following Treatment of an Intertrochanteric Hip Fracture With a Cephalomedullary Nail

**DOI:** 10.1155/2024/5590091

**Published:** 2024-08-21

**Authors:** Nathan C. Beckett, Jack Haglin, Paul Van Schuyver, Mark J. Spangehl, Maziyar A. Kalani, Mark K. Lyons, Abhijith R. Bathini, Joshua S. Bingham

**Affiliations:** ^1^Department of Orthopedic Surgery, Mayo Clinic, Phoenix, Arizona, USA; ^2^Department of Neurosurgery, Mayo Clinic, Phoenix, Arizona, USA

## Abstract

**Introduction:** Surgical management of intertrochanteric hip fractures is a common surgery with low rates of intraoperative complications. Vascular injuries are exceptionally rare when placing an intramedullary nail without open reduction. There are very few reported cases of direct arterial injury and active bleed at the level of the distal interlocking screw following closed reduction and intramedullary nailing of a hip fracture. We report one such case.

**Case Presentation:** An 88-year-old female presented to the emergency department with a left intertrochanteric hip fracture. Closed reduction with a cephalomedullary nail fixation of the left hip fracture occurred as planned without any obvious intraoperative technical issues. The patient remained stable intraoperatively. No open reduction was required. Postoperatively, the patient developed hemorrhagic shock and required massive transfusion protocol. Angiography demonstrated an intramuscular hematoma at the level of the distal intramedullary nail interlocking screw with active extravasation. The patient subsequently required embolization. Nine days following surgery, she began Eliquis for DVT prophylaxis and was ambulating independently with signs of hematoma resolution.

**Discussion:** Profunda femoris artery injury can stem from various mechanisms during surgery. Atherosclerosis places patients at a higher risk of complication due to rigid vessels. In this case, it is believed that drilling beyond the medial femoral cortex led to the arterial injury.

**Conclusion:** Care should be taken to prevent drills from plunging beyond the medial femoral cortex during surgery. Cautious observation of patient's vitals and clinical course can allow for early detection of vascular complication.

## 1. Introduction

### 1.1. Anatomy

The femoral artery is the primary blood supply to the lower extremities. It is a branch of the external iliac artery, which comes from the bifurcation of the common iliac artery. The femoral artery bifurcates into two main branches: the superficial femoral artery and the deep femoral artery, or profunda femoris artery. While the superficial femoral artery provides blood flow to the tissue below the knee, the profunda femoris artery is responsible for blood supply to the femur along with surrounding tissue [1]. The proximal branches of the profunda femoris arteries are the medial and lateral circumflex femoral arteries. As it continues distal, one or more branches from the profunda femoris artery supply the inner 2/3 of the cortex and the bone marrow of the femur [1]. The profunda femoris artery passes between the adductor longus and pectineus muscles, lying upon the pectineus, adductor brevis, and adductor magnus muscles in succession [2]. Its perforating branches pierce the adductor magnus tendon along the linea aspera [2].

Variations in the origin of the profunda femoris artery have been noted in the literature. Most commonly, it branches from the posterior aspect of the femoral artery, in the proximal one-third of the femur [3]. However, the posterior origin of the profunda femoris artery is only estimated to occur 39% of the time, with posterolateral origin being second most common and lateral origin being third [3]. The origin of the profunda femoris artery may correlate with the patient's ethnicity. For example, among Asian patients, the posterolateral origin of the profunda femoris artery was found to be most prominent [3]. Approximately 48% of profunda femoris arteries among all patient populations are estimated to arise from the upper third of the thigh, with 36% arising from the middle third, and 16% of profunda femoris arteries arising from the distal third of the thigh [3]. This variation is notable as it could affect orthopedic or interventional radiological procedures, reconstructive surgery, and other clinical and surgical processes.

### 1.2. Operative Fixation and Vascular Injury

Hip fracture is a prevalent musculoskeletal issue worldwide, with the rate of incidence estimated to be as high as 195 per 100,000 in the United States [4]. Hip fractures are more common among the elderly with a higher ratio of females to males, likely due to the increased risk of osteoporosis [4, 5]. Vascular injuries resulting from hip fracture surgery are rare. They have been estimated to occur at a rate of 0.25%–0.5% in all repairs and are thought to be much lower when open reduction is not required. As the population continues to age and the number of hip fractures continues to increase, it will become increasingly important to be aware of complications resulting from such surgeries [6, 7]. Most of these injuries are iatrogenic, and one study found less than 4% of vascular injuries secondary to hip fracture to have occurred prior to the surgery or in patients who did not receive surgery [7].

In 2015, Barquet, Gelink, and Giannoudis estimated that extrapelvic vessels were most commonly injured (91% compared to 8% for intrapelvic vessels). Among extrapelvic vessels, the artery at highest risk was the profunda femoris artery, which comprised 78% of extrapelvic vessel injuries [7]. The superficial femoral artery comprised 11% of the extrapelvic vessels injured [7]. There are limited reports in the literature reporting hematoma and pseudoaneurysm in the case of a closed reduction with placement of a short intramedullary nail [8], and even more rarely are cases seen involving a direct injury of the profunda femoris artery that resulted in an active bleed where open reduction was not required [9]. Given the scarcity of prior literature documenting this rare occurrence, the purpose of this report was to highlight this unique complication and briefly review the literature regarding the topic.

## 2. Case Presentation

The presented patient is an 88-year-old female with a history of hypertension, stable abdominal aortic aneurysm, chronic kidney disease (CKD), polymyalgia rheumatica (not corticosteroid dependant), and gastroesophageal reflux disease (GERD) who suffered a ground-level fall at her home. Prior to this injury, she used a cane for long-distance community ambulation with no other assistive devices and was not on anticoagulants. She functioned independently and lived with family. Of note, she suffered an intertrochanteric right femur fracture 8 years previously, managed with an intramedullary nail, and followed an uncomplicated postoperative course.

**Figure 1:**
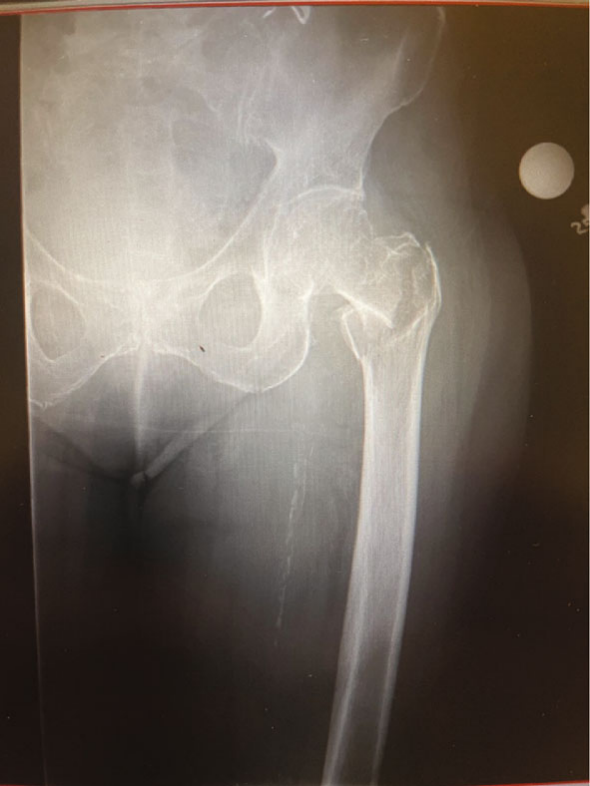
Left hip intertrochanteric fracture preoperation.

**Figure 2:**
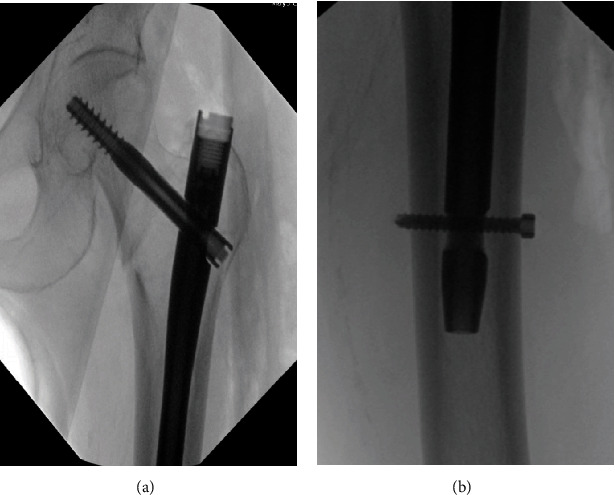
(a) Proximal femur immediately posthardware placement. (b) Distal femur at the level of the interlocking screw, immediately posthardware placement.

The patient presented to the emergency department with left hip pain and was found to have an isolated left hip intertrochanteric fracture (see Figure 1). She was subsequently taken to the operating room for closed reduction and fixation with a short cephalomedullary nail. The surgery was performed as planned, and the nail, lag screw, and distal interlocking screw were all confirmed to be placed properly via intraoperative fluoroscopy (see Figure 2). Traction was released following the successful placement of the distal interlocking screw, and the surgery was completed uneventfully. The patient remained stable while in the operating room and was extubated and taken to the recovery room without issue.

**Figure 3:**
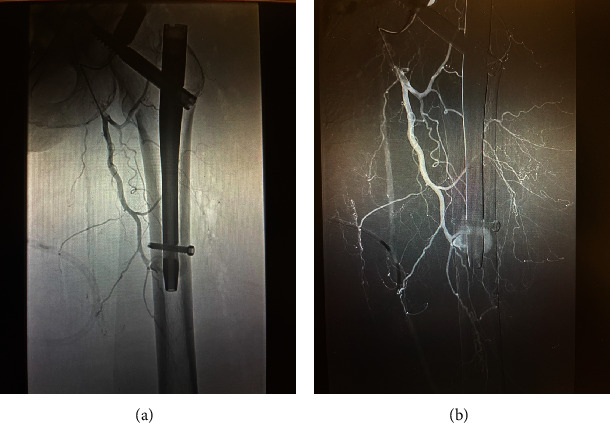
(a) Fluoroscopy demonstrating branch of the profunda femoris artery bleeding and hematoma. (b) Angiography view further demonstrating the bleeding branch of the profunda femoris artery.

That evening, upon return to the floor, she became hypotensive with systolic blood pressure down to the low 70 s mmHg. Her hemoglobin (Hgb) dropped to 8.5 from 11.5 mg/dL preoperatively. She was tachycardic, lethargic, and pale and felt increased left leg pain and swelling. Her mean arterial pressure (MAP) dropped below 60 mmHg. Our largest concern based on these symptoms was a postsurgical bleed. While compartment syndrome could also be a devastating sequela of swelling after arterial injury, the patient's swelling was not severe enough to cause this, and the patient had soft compartments on examination; thus, compartment syndrome was not an immediate concern in this case. After consultation with critical care, the patient was transferred to the ICU whereupon a massive transfusion protocol was activated, and the patient received three units of packed red blood cells for postoperative hemorrhagic shock. At this point, subsequent angiography was performed, and she was found to have active arterial bleeding from a branch of the profunda femoris artery at the level of the distal interlocking screw with an accompanying intramuscular hematoma (see Figure 3). Interestingly, despite a normal coagulation profile, placement of a central venous catheter was attempted in the contralateral right groin, but the critical care team had difficulty placing the right central line due to large amounts of bleeding. The patient developed a right retroperitoneal hematoma in this area. Given the rarity of this scenario, the critical care team gave a strong recommendation to emergently take the patient to the operating room for exploration of the bleeding vessel despite it being located on the medial midthigh, far from any incisions. After much multidisciplinary discussion, interventional radiology agreed to proceed with a successful embolization of the vessel. Pharmacological anticoagulation was held at this point.

Four days postoperatively, a large deep vein thrombosis was found in the contralateral thigh after an investigation of swelling in her nonoperative leg where the prior central catheter had been placed. An IVC filter was placed, and she was observed off anticoagulation as her serial Hgb and hematocrit testing had been stable. After she showed stability for over 24 h following IVC filter placement, heparin was started.

Six days following surgery, crackles were observed in her right chest. A chest X-ray showed an aspiration pneumonia for which she was placed on antibiotics. The following day (postoperative day seven), her Hgb dropped from 8.4 to 7 mg/dL. CT abdomen and pelvis showed no further sign of bleeding, and she responded well to one unit of packed red blood cells with her Hgb rising to 9.4 mg/dL. Nine days following surgery, the patient was transitioned from heparin to Eliquis, which was chosen as her Hgb and hematocrit were stable and she had been on Eliquis previously. At the time of discharge, she was ambulating independently, and the hematoma showed signs of resolution. At her last follow-up, 10 weeks postoperatively, she was doing well and recovering without further complications.

## 3. Discussion

There are several proposed mechanisms of arterial injury following hip fracture surgery. One of the most frequent etiologies is a fragment fracture of the lesser trochanter that causes injury either pre-, post-, or intraoperatively [7, 10]. An overshot drill bit or protruding screw past the medial cortex is the next most common cause [7]. Other listed injury mechanisms include overly forceful traction or vulnerable positioning, inappropriate arterial ligation, retractors, nail guide wires, and hooks [6–8, 11]. It should be noted that no difference in union or complication rates has been found between short and long intramedullary nails [12], and only one length of the short nail is used for intertrochanteric fracture repair; thus, nail length was not a factor in this case. In addition, Barquet et al. noted that vascular injuries from hip fracture repair surgery could be the same regardless of which mechanism caused them and that the same mechanism could result in diverse injuries. One commonality among causes and injury types was the risk factor of atherosclerosis that led to more rigid, low-flow arteries and a higher prevalence of injury [5]. Additionally, variation in the location of arterial branching and course could contribute to injury risk [3].

In our presented case, it is believed that while drilling the hole for the distal locking screw, the drill grabbed muscle tissue as it exited the medial bony cortex, pulling a branch of the profunda femoris artery near the femur and compromising the vessel. Alternatively, though no significant protrusion of the drill past the medial cortex was noted, the proximity of the vessel to the medial cortex combined with some penetration past the cortex may have resulted in injury to the vessel. However, because the patient's vitals were steady and satisfactory during the surgery, the complication was not noted until her return to the floor. This report is meant to highlight the occurrence of this rare complication. Further, we recommend engaging interventional radiology as soon as possible in the case of suspected bleed, so that embolization may be performed without delay if needed. We also recommend resuming anticoagulation after embolization despite the injury, as our patient developed a large DVT requiring an IVC filter due to concerns of bleeding with prophylactic anticoagulation.

## 4. Conclusion

Albeit rare, iatrogenic vascular injury does occur during hip fracture repair. Care should be taken to prevent the drill from passing the medial femoral cortex for any distal locking screws. Atherosclerotic disease and individual variation of arterial anatomy could increase the risk of surgical injury. Monitoring Hgb levels and blood pressure and careful observance for abnormal swelling or pain following surgery can assist providers in detecting complications early. Resuming anticoagulation management as soon as it is safe following surgery may prevent further issues such as deep vein thrombosis.

## Data Availability

There is no further relevant data outside what was described in this case report. The identity of the subject of this case report will remain anonymous to observe patient confidentiality.
